# Let There Be Light: Genome Reduction Enables *Bacillus subtilis* to Produce Disulfide-Bonded *Gaussia* Luciferase

**DOI:** 10.1021/acssynbio.3c00444

**Published:** 2023-11-27

**Authors:** Tobias Schilling, Borja Ferrero-Bordera, Jolanda Neef, Sandra Maaβ, Dörte Becher, Jan Maarten van Dijl

**Affiliations:** † Department of Medical Microbiology, University of Groningen, University Medical Center Groningen, Hanzeplein 1, P.O. Box 30001, 9700RB Groningen, The Netherlands; ‡ Institute of Microbiology Department of Microbial Proteomics, University of Greifswald, D-17489 Greifswald, Germany

**Keywords:** Bacillus subtilis, genome reduction, recombinant protein production, proteolysis, disulfide bond formation, *Gaussia* Luciferase

## Abstract

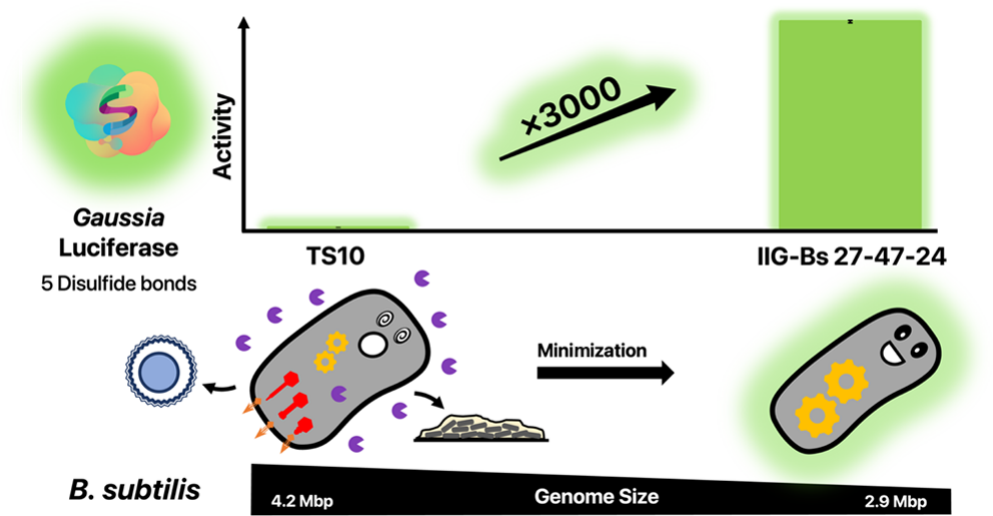

*Bacillus subtilis* is a major workhorse for enzyme production in industrially relevant quantities. Compared to mammalian-based expression systems, *B. subtilis* presents intrinsic advantages, such as high growth rates, high space-time yield, unique protein secretion capabilities, and low maintenance costs. However, *B. subtilis* shows clear limitations in the production of biopharmaceuticals, especially proteins from eukaryotic origin that contain multiple disulfide bonds. In the present study, we deployed genome minimization, signal peptide screening, and coexpression of recombinant thiol oxidases as strategies to improve the ability of *B. subtilis* to secrete proteins with multiple disulfide bonds. Different genome-reduced strains served as the chassis for expressing the model protein *Gaussia* Luciferase (GLuc), which contains five disulfide bonds. These chassis lack extracellular proteases, prophages, and key sporulation genes. Importantly, compared to the reference strain with a full-size genome, the best-performing genome-minimized strain achieved over 3000-fold increased secretion of active GLuc while growing to lower cell densities. Our results show that high-level GLuc secretion relates, at least in part, to the absence of major extracellular proteases. In addition, we show that the thiol–disulfide oxidoreductase requirements for disulfide bonding have changed upon genome reduction. Altogether, our results highlight genome-engineered *Bacillus* strains as promising expression platforms for proteins with multiple disulfide bonds.

## Background

Various bacteria of the genus *Bacillus* have been used extensively as biotechnological production platforms for recombinant proteins such as industrial enzymes, as well as antibiotics, insecticides, and fine chemicals.^1−4^*Bacillus subtilis* is one of the best-studied species from this genus, especially since it has served for many years as a model organism for Gram-positive bacteria in general. Consequently, a high number of mutant strains and molecular tools are presently available for fundamental and applied research on *B. subtilis*.

As *B. subtilis* thrives naturally mainly in the soil and plant rhizosphere where it has evolved to utilize a very wide spectrum of different substrates, this bacterium secretes many macromolecule-degrading enzymes into its extracellular environment.^5,6^ These include amylases, arabinases, chitinases, mannanases, cellulases, xylanases, as well as a range of proteases required for nutrient acquisition and protein quality control.^5^ As the secretion capabilities of *B. subtilis* have evolved well due to natural selection pressures, it can also deliver high yields of biotechnologically relevant enzymes in industrially optimized processes, which may amount well over 25 g/L culture.^7^ These enzymes are mostly secreted via the general protein secretion (Sec) pathway, although alternative secretion routes exist as well.^8−11^

Eight exoproteases of *B. subtilis* have been described previously. These areNprB (UniProt ID: P39899), AprE (UniProt ID: P04189), Epr (UniProt ID: P16396), Bpr (UniProt ID: P16397), NprE (UniProt ID: P68736), Mpr (UniProt ID: P39790), Vpr (UniProt ID: P29141), and WprA (UniProt ID: P54423). While naturally secreted exoproteases of bacilli, like subtilisin, are successfully deployed in detergents, cosmetics, food processing, and organic chemistry, there is also a downside to them since they may (i) degrade secreted proteins of interest (POIs) and (ii) interfere with beneficial cell wall-associated proteins involved in protein quality control.^12,13^ Both of these effects can lower the potential yield of secreted POIs significantly. However, since these exoproteases are dispensable for the growth of *B. subtilis* under controlled culture conditions with optimized nutrient supply, deletion of their genes from the genome has been recently applied as a common procedure for strain optimization.^14,15^ In addition, industrially relevant *Bacillus* strains are also required to be (i) sporulation-deficient to avoid spore formation in a bioreactor, and (ii) cured from prophages which can cause severe autolysis upon bioproduction stress.^16^ Besides such targeted gene deletions, also large-scale genome reduction has been explored to further increase the capabilities of *B. subtilis* as a protein production platform.^17−20^ In particular, this has led to the midi- and mini*Bacillus* strain line.^16,21−23^ While these genome-reduced strains provided valuable insights on the minimal essential gene set for a robust living organism, some of them showed significantly increased yields of notoriously “difficult-to-produce proteins”.^17^ These findings indicate that genome-reduced strains may offer huge advantages for recombinant protein production.

The production of disulfide-bonded proteins imposes a specific requirement on bacterial cell factories, namely, the oxidation of cysteine thiols. Because the cytoplasm of living cells is a generally reducing environment, thiol oxidation takes place mostly within the endoplasmic reticulum of eukaryotes or extracytoplasmic compartments of bacteria such as the membrane–cell wall interface of Gram-positive bacteria and the periplasm of Gram-negative bacteria. Disulfide bond formation within secretory proteins is most extensively encountered in eukaryotes, while prokaryotes employ this post-translational modification less extensively for protein folding and stabilization. Consequently, disulfide bond formation can represent a serious bottleneck in the expression of numerous eukaryotic proteins in bacteria. Although thiol oxidation can happen spontaneously, in living cells, this process is usually catalyzed by thiol–disulfide oxidoreductases (TDORs). Such enzymes are conserved across many species, including *B. subtilis*, where the TDORs BdbC and BbdD have a major role in the secretion of disulfide-bonded proteins.^24^ To enhance the capabilities of *B. subtilis* for disulfide bond formation, the integration of recombinant thiol oxidases, especially the staphylococcal thiol oxidase DsbA, in combination with reduced expression of the main disulfide reductase Thioredoxin A (TrxA) was previously explored.^25^ This led to a 3.5-fold increase in the yield of a secreted alkaline phosphatase A (PhoA) from *Escherichia coli*, which has two disulfide bonds that are required for stability and activity.

The efficiency of protein secretion in *Bacillus* depends on diverse parameters, including the properties of the POI and the signal peptide (SP) used to direct it into the Sec pathway,^26,27^ the type of promoter used for expression,^28−31^ the cultivation conditions,^32^ the modulation of particular chaperones and secretion pathway components,^33−35^ or combinations thereof.^36,37^ Although machine learning approaches to select optimal SPs in silico are currently being developed,^38,39^ the identification of suitable SPs still requires experimental testing in the laboratory.

So far, several eukaryotic proteins have been produced in *Bacillus*,^40,41^ and most of them required disulfide bond formation. While the spectrum of antibodies expressed in *B. subtilis* remains small to date, expression of the chicken egg lysozyme-binding single-chain antibody (scFv) D1.3 showed promising yields of around 120 mg of active antibody per liter culture.^42^ Moreover, protease-deficient strains were shown to be versatile and stable platforms for the production of single-domain antibodies (also known as nanobodies) with yields of 15 to 20 mg of nanobodies per liter of culture medium in a nonoptimized process.^43^ A combination of the aforementioned optimization parameters allowed secretory production of the human growth factor hFGF21.^43^ However, the overall product yield was still rather low, presumably due to limitations in the disulfide bond formation. Clearly, an easy-to-monitor disulfide-bonded model protein would simplify the benchmarking of different strains for their ability to catalyze disulfide bond formation. One such protein is the luciferase from the bioluminescent copepod *Gaussia princeps* (GLuc),^44^ which has been used as a reporter molecule in mammalian cells and *E. coli*.^45^ The structure and biochemical properties of GLuc were recently resolved,^46^ showing that this protein of 168 amino acid residues contains five disulfide bonds.

To date, it was not known whether a protein with more than two disulfide bonds can be effectively produced by *B. subtilis*, and whether genome-reduced *B. subtilis* strains would excel for this purpose as was recently shown for other difficult-to-produce proteins.^47^ The present study was therefore aimed at determining the effect of large-scale genome reduction in *B. subtilis* 168 on the secretory production of GLuc. For benchmarking, we compared the expression of GLuc to that of *E. coli* PhoA.

## Methods

### Media and Solutions

All media and solutions were prepared using water processed with a Milli-Q Direct Water Purification System (Merck KGaA, Darmstadt, Germany) and sterilized by autoclaving at 121 °C for 15 min. Heat-sensitive medium additives were filter-sterilized.

#### Lysogeny Broth (LB)

If not otherwise stated, *E. coli* and *B. subtilis* strains were cultured in LB (10 g/L tryptone, 10 g/L NaCl, and 5 g/L yeast extract).^30^ LB agar contained 1.5% (w/v) Agar–Agar.

#### 2× Tryptone-Yeast (TY) Medium

2× TY medium contained 10 g/L tryptone, 6 g/L yeast extract, and 0.9 g/L CaCl_2_·7H_2_O, adjusted to pH 6.8.

#### Spizizen Medium

The medium used in this study was modified from the original version described by Spizizen.^48^ 1 L of 2× Spizizen medium was prepared by adding 28 g of K_2_HPO_4_, 12 g of KH_2_PO_4_, 4 g of l-glutamate, 2.3 g of Na_3_-citrate·2H_2_O, and 0.4 g of MgSO_4_·7H_2_O to 900 mL of water. The pH was adjusted to pH 7.0 with 10 M NaOH. Subsequently, water was added to a final volume of 1 L, and the medium was sterilized by autoclaving. 1× Spizizen-plus medium was prepared by mixing 10 mL of 2× Spizizen medium, 9.56 mL of water, 200 μL 50% [w/v] glucose, 20 μL of tryptophane (2 mg/mL stock solution), 200 μL of casamino acids (2% [w/v] stock solution), and 20 μL of ferric ammonium citrate (2.2 mg/mL stock solution). 1× Spizizen-starvation medium was prepared by mixing 10 mL of 2× Spizizen medium, 9.8 mL of water, and 200 μL of 50% glucose.

#### Antibiotics

Unless stated otherwise, media for strains carrying antibiotic resistance markers were supplemented with 100 mg/L ampicillin for *E. coli*, 150 mg/L erythromycin for *E. coli*, 50 mg/L (for *E. coli*) or 25 mg/L (for *B. subtilis*) kanamycin, 10 mg/L chloramphenicol for *B. subtilis*, or 10 mg/L tetracycline for *B. subtilis*.

### Strain Maintenance

For protein expression studies, four *B. subtilis* chassis strains were used: TS10, IIG-Bs27-31, IIG-Bs27-39, and IIG-Bs27-47-24 (Table 1), each carrying either a GLuc- or PhoA-encoding plasmid or no plasmid as a control (Table 1). Unless stated differently, all *B. subtilis* strains were cultured at 37 °C and with vigorous shaking at 250 rpm, in 20 mL of medium, using 250 mL baffled glass shake flasks (Carl Roth GmbH & Co. kg, Karlsruhe, Germany). For general strain maintenance, the bacteria were grown in LB medium. *E. coli* cells were grown at 37 °C with vigorous shaking at 250 rpm, in 10 mL of LB medium, in 100 mL nonbaffled shake flasks.

**Table 1:** Strains, Plasmids, and Antibodies Used in This Study

strain	genotype	phenotype	reference
*B. subtilis* BSB1	*B. subtilis* 168 carrying the *trpC* gene from *B. subtilis* HVS495	tryptophane prototroph	(49)
*B. subtilis* TS10	168 *trpC* Δ*yvcA*::P_*mtl*_-*comKS*, 4.2 Mbp	prototroph, supercompetent	this study
*B. subtilis* IIG-Bs27-31	genome-reduced to 3.4 Mbp	deficient in sporulation, exoproteases, and prophages	(23)
*B. subtilis* IIG-Bs27-39	genome-reduced to 3.3 Mbp	higher biomass formation and growth rate	(23)
*B. subtilis* IIG-Bs27-47-24	genome-reduced to 2.9 Mbp	lower growth rate, unable to grow in most defined media, shows higher secretion yields for some proteins	(23)
*B. subtilis* BRB01	168 Δ*nprB*	protease-deficient	(14)
*B. subtilis* BRB02	168 Δ*nprB* Δ*aprE*	protease-deficient	(14)
*B. subtilis* BRB03	168 Δ*nprB* Δ*aprE* Δ*epr*	protease-deficient	(14)
*B. subtilis* BRB04	168 Δ*nprB* Δ*aprE* Δ*epr* Δ*bpr*	protease-deficient	(14)
*B. subtilis* BRB05	168 Δ*nprB* Δ*aprE* Δ*epr* Δ*bpr* Δ*nprE*	protease-deficient	(14)
*B. subtilis* BRB06	168 Δ*nprB* Δ*aprE* Δ*epr* Δ*bpr* Δ*nprE* Δ*mpr*	protease-deficient	(14)
*B. subtilis* BRB07	168 Δ*nprB* Δ*aprE* Δ*epr* Δ*bpr* Δ*nprE* Δ*mpr* Δ*vpr*	protease-deficient	(14)
*B. subtilis* BRB08	168 Δ*nprB* Δ*aprE* Δ*epr* Δ*bpr* Δ*nprE* Δ*mpr* Δ*vpr* Δ*wprA*	protease-deficient	(14)

plasmids	relevant genotype and/or relevant characteristics	parental plasmid	source
pBSMul1	pUB110 ori for replication in *B. subtilis*, *ampR* (for *E. coli*), *kanR* (for *Bacillus*)	pMA5	(26)
pBSMul1_GLuc	GLuc gene	pBSMul1	this study
pBSMul1_SP_EPR__GLuc	SP_epr_ signal peptide	pBSMul1	this study
pBSMul1_SP_EPR+1__GLuc	SP_epr+1_ signal peptide	pBSMul1_ GLuc	this study
pBSMul1_SP_EPR+2__GLuc	SP_epr+2_ signal peptide	pBSMul1_ GLuc	this study
pBSMul1_SP_EPR+3__GLuc	SP_epr+3_ signal peptide	pBSMul1_ GLuc	this study
pBSMul1_SP_wapA__GLuc	SP_wapA_ signal peptide	pBSMul1_ GLuc	this study
pBSMul1_SP_wapA+1__GLuc	SP_wapA+1_signal peptide	pBSMul1_ GLuc	this study
pBSMul1_SP_wapA+2__GLuc	SP_wapA+2_ signal peptide	pBSMul1_ GLuc	this study
pBSMul1_SP_wapA+3__GLuc	SP_wapA+3_ signal peptide	pBSMul1_ GLuc	this study
pBSMul1_SP_sacB__GLuc	SP_sacB_ signal peptide	pBSMul1_ GLuc	this study
pBSMul1_SP_sacB+1__GLuc	SP_sacB+1_ signal peptide	pBSMul1_ GLuc	this study
pBSMul1_SP_sacB+2__GLuc	SP_sacB+2_ signal peptide	pBSMul1_ GLuc	this study
pBSMul1_SP_sacB+3__GLuc	SP_sacB+3_ signal peptide	pBSMul1_ GLuc	this study
pBSMul1_SP_yncM__GLuc	SP_yncM_ signal peptide	pBSMul1_ GLuc	this study
pBSMul1_SP_yncM+1__GLuc	SP_yncM+1_ signal peptide	pBSMul1_ GLuc	this study
pBSMul1_SP_yncM+2__GLuc	SP_yncM+2_ signal peptide	pBSMul1_ GLuc	this study
pBSMul1_SP_yncM+3__GLuc	SP_yncM+3_ signal peptide	pBSMul1_ GLuc	this study
pBSMul1_SP_tasA__GLuc	SP_tasA_ signal peptide	pBSMul1_ GLuc	this study
pBSMul1_SP_tasA+1__GLuc	SP_tasA+1_ signal peptide	pBSMul1_ GLuc	this study
pBSMul1_SP_tasA+2__GLuc	SP_tasA+2_ signal peptide	pBSMul1_ GLuc	this study
pBSMul1_SP_tasA+3__GLuc	SP_tasA+3_ signal peptide	pBSMul1_ GLuc	this study
pPSPhoA5	SP-Pro-lip_PhoA, *cmR*	pPS2	(50)
pJOE9658	ΔP_*manPA*_::P_*tetLM*_, *kanR* (for *E. coli*)	pJOE8999	(51)
pTS102	sgRNA + HR template for Δ*yvcA*::P_*mtl*_-*comKS* insertion	pJOE9658	this study

antibodies	specific binding target	type	source
Invitrogen PA1-181	*Gaussia* Luciferase	Rabbit, polyclonal	Thermo Fisher Scientific
Custom Rabbit Serum	*B. subtilis* TrxA	Rabbit, polyclonal	Euro-gentec
Sigma-Aldrich MAB1012	*E. coli* PhoA	Mouse, monoclonal	Merck KGaA
IRDye 800CW Goat anti-Rabbit IgG	Rabbit IgG	Goat, IRDye 800CW conjugated	LI-COR Biosciences GmbH (Bad Homburg, Germany)
IRDye 800CW Goat anti-Mouse IgG	Mouse IgG	Mouse, IRDye 800CW conjugated	LI-COR Biosciences GmbH

### Transformation of *B. subtilis*

To create the *B. subtilis* strain TS10, a supercompetence cassette was inserted into the genome of *B. subtilis* BSB01. To this end, 168 *trp+* cells were transformed with the CRISPR-Cas9 plasmid pTS102, using a modified method after Spizizen.^48^ The bacteria were grown in 2xTY medium overnight and diluted to an OD_600_ of 0.1 in 1× Spizizen-plus medium. After growth for 2–3 h to an OD_600_ ≈ 0.4 to 0.6, the culture was diluted 1:1 with 1× Spizizen-starvation medium in a 500 mL shake flask and incubated for another 1.5 h. The culture was centrifuged at 3000*g* in a tabletop centrifuge for 10 min at room temperature. 90% of the supernatant was removed, and the pellet was resuspended in the remainder. Aliquots of 500 μL cells were mixed with 100 ng of plasmid DNA in a 15 mL Falcon tube and incubated for 1 h. Subsequently, 0.5 mL of LB medium was added and incubated for 1 h. The cells were pelleted and plated onto LB agar plates with selective antibiotics. The CRISPR-Cas9-mediated modification of strain BSB01 was carried out as described by Toymentseva and Altenbuchner.^51^

The strains TS10, IIG-Bs27-31, IIG-Bs27-39, and IIG-Bs27-47-24 were transformed making use of the introduced supercompetence cassette as described by Rahmer et al.^52^

### Plasmid Construction

The CRISPR-Cas9 plasmid pTS102 was created based on pJOE9658,^51^ as described by Schilling et al.^53^ The primers TS101 and TS102 were used for spacer sequence insertion resulting in pTS101, primers TS103 and TS104 for PCR amplification of the supercompetence cassette including flanks for homologous recombination from gDNA of IIG-Bs-27-47-24, and TS105 and TS106 for amplification of pTS101 to insert the supercompetence cassette.

The plasmid pBSMul1_GLuc was prepared by assembly cloning of three PCR fragments using the Phusion High-Fidelity DNA Polymerase, the NEBuilder HiFi DNA Assembly Cloning Kit, and NEB 10-β Competent *E. coli* cells (all from New England Biolabs, Ipswich, Massachusetts), following the protocol of the manufacturer. For constructing the mother plasmid pBSMul1_GLuc, the plasmid backbone was amplified from pBSMul1^26^ using the primers pBSMul1.fw and pBSMul1.rev, and the GLuc-encoding gene from a synthetic DNA fragment (GenBank Accession Number AY015993.1, not codon-optimized, excluding the native SP as annotated with SignalP^54^) using the primers GLuc_JN.fw and GLuc_JN.rv. The SP-encoding plasmids based on the mother plasmid pBSMul1_GLuc were constructed by assembly cloning as well, while all SP-encoding sequences were amplified from gDNA of *B. subtilis* 168. To create pBSMul1_SP_EPR__GLuc, the backbone was amplified from pBSMul1_GLuc using the primers pBSMul.fw and epr-GLucJN.fw, and the Epr SP from *B. subtilis* 168 gDNA using the primers eprJN.fw and eprJN.rv. The remaining plasmids encoding other SPs were created in the same manner. To create, for example, pBSMul1_SP_epr+1__GLuc, the backbone was amplified from pBSMul1_GLuc using the primers pBSMul.fw and epr-GLuc+1AAJN.fw, and the Epr+1 SP-encoding sequence from *B. subtilis* 168 gDNA using the primers eprJN.fw and epr+1AAJN.rv. Sequences of the obtained plasmids were verified via Sanger Sequencing using the primers pBSMulIcol.fw and pBSMulIcol.rev.

All oligonucleotides used as PCR primers are listed in Supporting Table S1.

Plasmids were isolated from overnight cultures of *E. coli* using the innuPREP Plasmid Mini Kit (Analytik Jena AG, Jena, Germany).

### Protein Production Experiments

For protein production experiments, the *B. subtilis* strains were grown in 20 mL of LB medium with the respective antibiotics added. Precultures were inoculated from single colonies from LB agar plates that had been incubated overnight and grown for 16 h. Main cultures were grown for 18–20 h. After harvesting, the cultures were chilled and kept on ice.

### Protein Analysis

For the analysis of cellular and secreted proteins, cultures were normalized to 1 mL of culture with an OD_600_ of 2. A respective volume of each culture was centrifuged at 14,000*g* for 2 min at 4 °C in a 2 mL screw-cap tube, and the culture supernatant fraction with secreted proteins was transferred to a new tube. The pelleted cells were disrupted by adding 200 μL of 1× NuPAGE lithium dodecyl sulfate (LDS) Sample Buffer (including NuPAGE Sample Reducing Agent) (Thermo Fisher Scientific, Waltham, Massachusetts) and a spatula tip of glass beads with 0.1 mm diameter (Scientific Industries Inc., Bohemia, New York), followed by 2 min of bead-beating in a Precellys24 tissue homogenizer. The sample was subsequently heated to 70 °C for 10 min, briefly centrifuged, and the supernatant was carefully transferred to a new tube.

Extracellular proteins in culture supernatant fractions were precipitated with trichloroacetic acid (TCA) to increase their concentrations for further analysis. To this end, the cultures were normalized to 1 mL of culture with an OD_600_ = 2. A respective volume of each culture was diluted with 1× PBS buffer to a volume of 1.2 mL, to which 300 μL of 50% [v/v] TCA were added. After incubation for 15 min at −20 °C, samples were centrifuged at 14,000*g* in a tabletop centrifuge for 20 min at 4 °C. The liquid was removed and exchanged with 800 μL of cold acetone stored at −20 °C. After centrifuging again at 14,000*g* for 20 min at 4 °C, the acetone was carefully removed using a pipet tip, and the residual liquid was evaporated in a vacuum centrifuge. The dried protein pellets were resuspended in 200 μL of 1× NuPAGE LDS Sample Buffer (including NuPAGE Sample Reducing Agent).

For LDS-PAGE, NuPAGE Bis-Tris Midi Gels were loaded with 10 μL of a protein sample. Electrophoresis was performed for 1 h, 160 V constant, and maximum 200 mA. For direct visualization of the separated proteins, gels were stained with InstantBlue Coomassie Protein Stain (Abcam, Cambridge, U.K.).

For Western blotting, the proteins were transferred onto Amersham Protran Western blotting membranes using an Invitrogen Power Blotter System (Thermo Fisher Scientific). Membranes were incubated in 5% skim milk solution in 1× PBS-Tween buffer overnight and washed thoroughly in 1× PBS-Tween. For protein detection, the membrane was incubated for 1 h with a specific primary antibody diluted 1:5000 in 1× PBS-Tween, and subsequently, it was washed thoroughly in 1× PBS-Tween. Subsequently, the membrane was incubated for 1 h with a fluorescently labeled secondary antibody diluted 1:5000 in 1× PBS-Tween, and subsequently washed thoroughly in 1× PBS. All antibodies used are listed in Table 1. Detection of fluorescent signals was performed using an Amersham Typhoon biomolecular imager (Danaher, Washington, DC).

### *Gaussia* Luciferase Activity Assay

The *Gaussia* luciferase activity assay was performed using a Pierce Gaussia Luciferase Glow Assay Kit (Thermo Fisher Scientific) according to the manufacturer’s protocol. As samples for this assay, 20 μL of cell-free culture supernatant, or pelleted bacteria resuspended in the original volume of cell lysis buffer (i.e., 10 mg/mL Lysozyme dissolved in 1× PBS buffer) were incubated with the reagent from the kit for 30 min at 37 °C. The measurement of luciferase activity was done in triplicate, using a Biotek Synergy 2 Multi-Detection microplate reader (Biotek Instruments, Winooski, Vermont).

### Alkaline Phosphatase Activity Assay

The assay to detect alkaline phosphatase activity was performed as described previously^55^ with modifications. 6 μL of sample was thoroughly mixed with 144 μL of a freshly prepared substrate solution (3.73 mM *p*-nitrophenyl phosphate [pNPP], 0.33 M diethanolamine, and 0.16 mM magnesium chloride, pH 9.8). Alkaline phosphatase activity was determined kinetically in triplicate by measuring the increase in optical density at 405 nm (OD_405_) for 30 min in 35 s intervals, with incubation at 37 °C and under constant shaking, using a Biotek Synergy 2 Multi-Detection microplate reader.

### Exoprotease Treatment of Culture Supernatant Samples

To test the possible degradation of secreted proteins by exoproteases of *B. subtilis*, 500 μL of cell-free culture supernatant from each investigated strain was incubated with an equal volume of cell-free culture supernatant of *B. subtilis* TS10 for 2 h at 37 °C. Subsequently, the samples were processed as described above, but the results were normalized according to the 2× dilution.

### DTT Treatment of GLuc

Protein samples were incubated for 2 h at room temperature in the presence of 10 mM dithiothreitol (DTT).

## Results

### Signal Peptide Screening for GLuc Secretion

**Figure 1:**
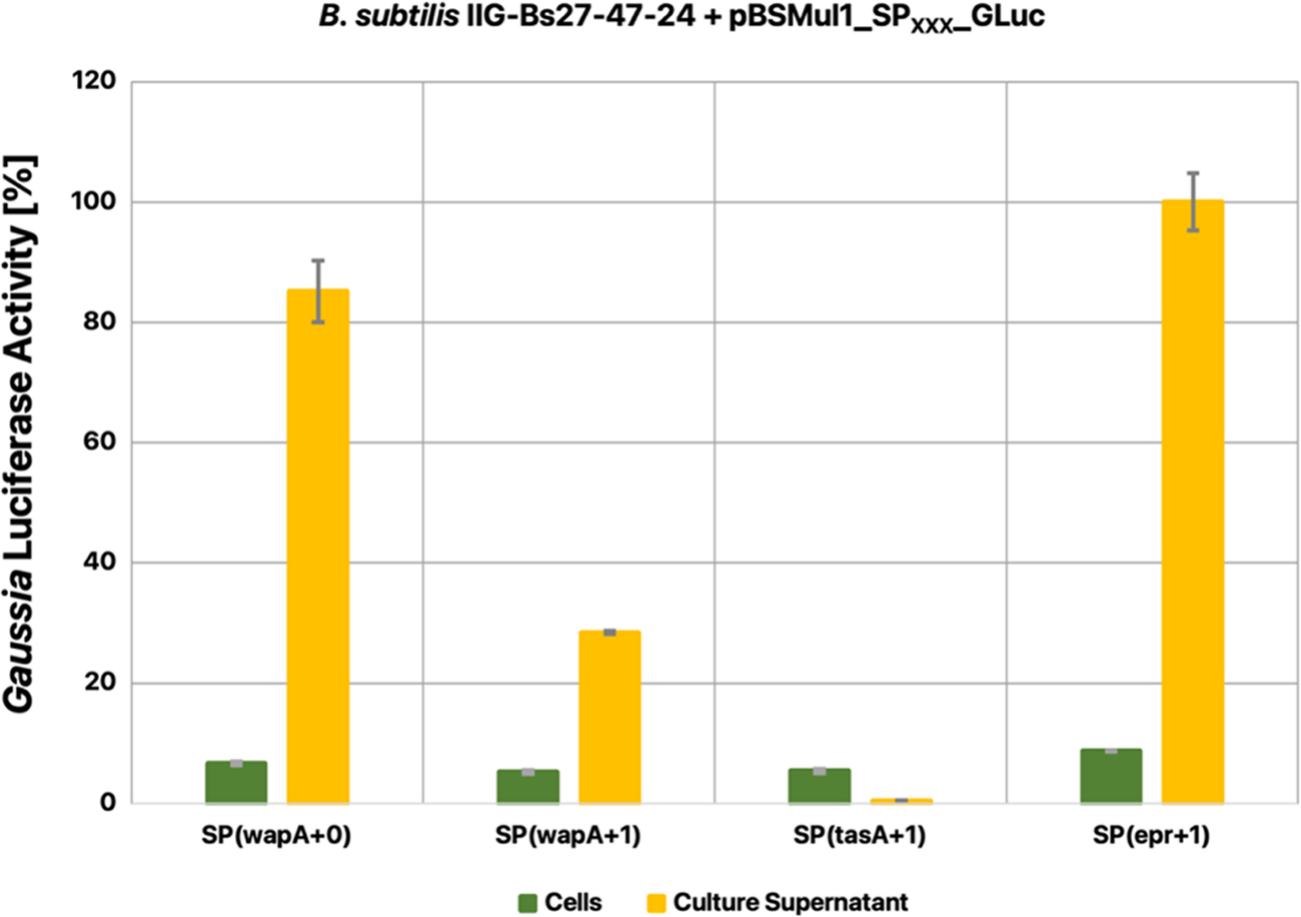
Signal peptide screening for GLuc secretion by *B. subtilis*. GLuc activities in the cell and culture supernatant fractions of *B. subtilis* IIG-Bs27-47-24 grown for 16 h in LB medium and producing GLuc with N-terminal signal peptide (SP) fusions as indicated were measured using the *Gaussia* Luciferase Glow Assay. The measured data in relative light units (see Supporting Table S2) was normalized, wherein the highest measured activity in the supernatant fraction of the bacteria producing GLuc fused to SP_epr+1_ was set to 100%.

To test whether *B. subtilis* can secrete the *Gaussia* luciferase, this protein was fused to the SPs of the secreted Epr (UniProt ID: P16396), SacB (UniProt ID: P05655), and TasA (UniProt ID: P54507), WapA (UniProt ID: Q07833), or YncM (UniProt ID: O31803) proteins of *B. subtilis*. In particular, we constructed fusions of each SP and the mature GLuc protein sequence either directly at the signal peptidase cleavage site of each SP (designated with +0), or at the first, second, or third amino acid residue of the mature Epr, SacB, TasA, WapA, or YncM proteins (designated with +1, + 2, or +3). Subsequently, the plasmids encoding the respective SP-GLuc fusions were introduced in the *B. subtilis* strain TS10 with a full-size genome, and the midi*Bacillus* strain IIG-Bs27-47-24, and the secretion of GLuc into the culture medium was inspected by LDS-PAGE and Western blotting (Supporting Figure S1). Interestingly, upon separation of the bacterial cells and culture medium by centrifugation, no GLuc could be detected in the culture supernatant of the TS10 strain. On the other hand, fusion of GLuc to the SP_wapA+0_, SP_wapA+1_, or SP_epr+1_ resulted in effective GLuc secretion by the genome-reduced strain IIG-Bs27-47-24, indicating that the fusion of this protein to an appropriate SP is of critical importance. Furthermore, all analyzed cell-fraction samples showed a basal level of GLuc activity independent of the SP that was fused to this protein. Based on these observations, we performed luciferase activity assays to measure the activity of the cell-associated and secreted GLuc. As shown in Figure 1, the SP_epr+1_ directed the highest level of active GLuc secretion. Therefore, this SP was selected for our further investigations on GLuc secretion in *B. subtilis*.

### Expression of Gaussia Luciferase in Different Genome-Minimized Strains

To determine to what extent different degrees of genome reduction would influence the yields of active secreted GLuc, we introduced the pBSMul1_SP_epr+1_-GLuc plasmid in four strains with different levels of genome reduction, namely, *B. subtilis* IIG-Bs27-31, IIG-Bs27-39, IIG-Bs27-47-14, and IIG-Bs27-47-24 (Table 1). The *B. subtilis* TS10 strain was included in these analyses as a control. To compare the expression of GLuc with that of another disulfide-bonded protein, we also introduced the pPSPhoA5 plasmid into these five strains, which allowed us to assess their ability to secrete *E. coli* PhoA.

**Figure 2:**
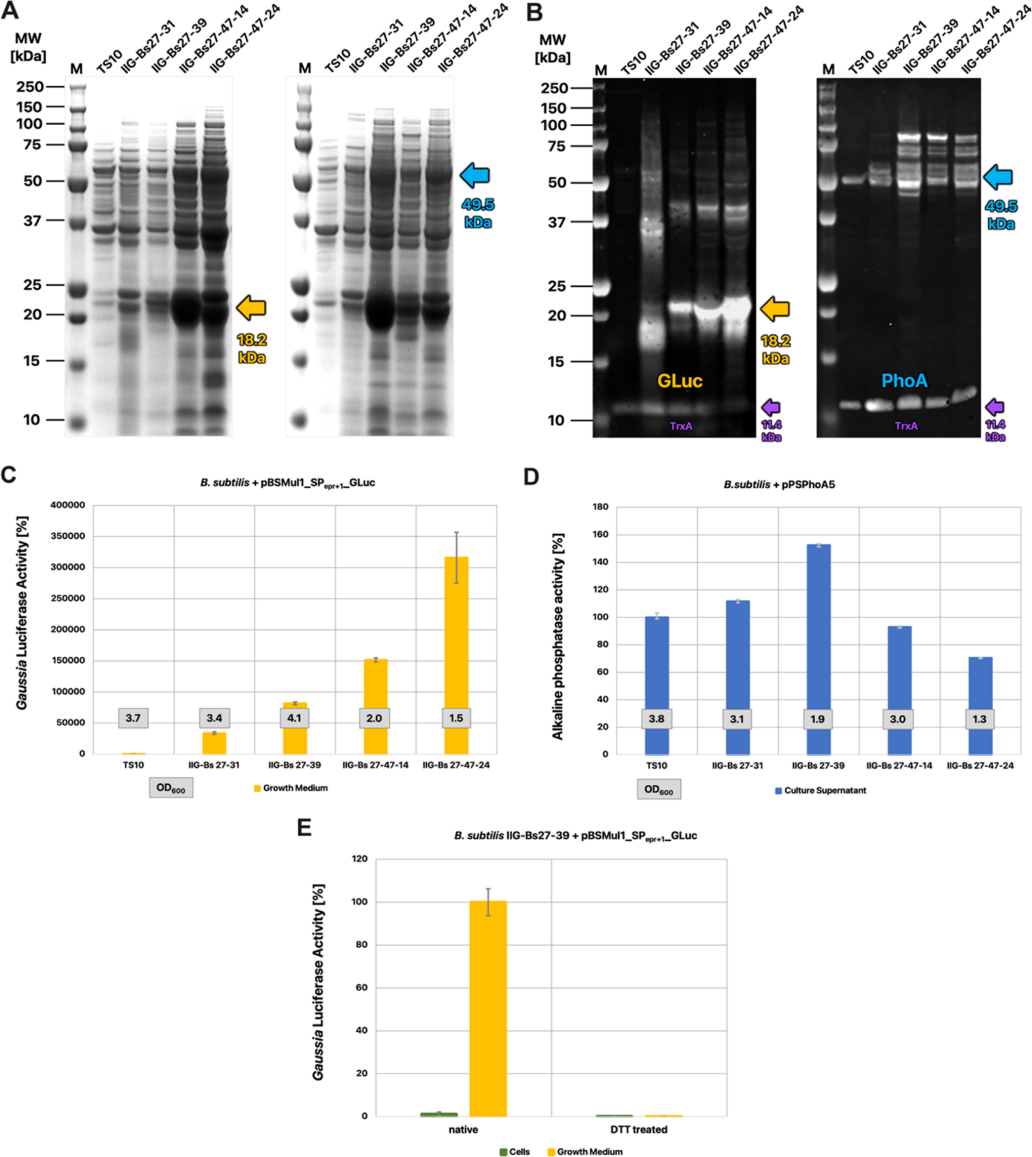
Benchmark of different genome-minimized strains producing GLuc or PhoA. (A, B) Culture supernatant samples from four different genome-minimized *B. subtilis* strains and the reference strain TS10, producing either GLuc (18.2 kDa) or PhoA (49.5 kDa), were separated by LDS-PAGE and analyzed by Coomassie staining (A) or Western blotting with specific antibodies against GLuc or PhoA (B), respectively. In addition, both Western blots were analyzed with an antibody against the cytoplasmic TrxA protein (11.4 kDa), which serves as a reliable marker for cell lysis (B) (for the control blots without TrxA detection, see Supporting Figure S2). The samples were normalized to the OD_600_ of the respective strain. The arrows indicate the expected molecular size in kDa of the respective mature proteins. (C, D) Comparison of the enzymatic activities of GLuc and PhoA per mL of culture supernatant sample as used in (A) and (B). The measured data in activity units (Supporting Table S2) was normalized, whereby the values measured for the reference strain TS10 were set to 100%. Boxed numbers indicate the respective OD_600_ of the expressing strain post fermentation. (E) Luciferase activity assay on cellular and culture supernatant samples from the GLuc-producing strain IIG-Bs27-39 before and after treatment with DTT. The measured data in relative light units (Supporting Table S2) was normalized, wherein the value measured for the native culture supernatant was set to 100%.

Analysis of the culture supernatant fractions of the different strains by LDS-PAGE and Western blotting revealed huge differences with respect to the quality and quantity of secreted GLuc, depending on the level of genome reduction (Figure 2A,B). While no significant amounts of GLuc were detectable in media of the different TS10-based strains, the GLuc secreted by genome-minimized strains was readily detectable on the Coomassie-stained gels. Two secreted GLuc products were detectable by Western blotting: a main product (∼20 kDa) occurring as distinct band, and a degradation product (<20 kDa) occurring as a nondistinct smear. With progressive genome reduction, the ratio of the main product and the degradation gradually increased, with the IIG-Bs27-47-24 strain showing the highest amounts of the main product.

To verify whether the increased amount of GLuc in the culture supernatant was not the result of cell lysis but rather actual secretion, we additionally inspected the GLuc Western blots with an antibody against TrxA, which is a cytoplasmic protein whose detection in the culture supernatant indicates cell lysis. As shown in Figure 2B, the TrxA signal decreased with progressive genome reduction, indicating decreasing levels of cell lysis.

In contrast to GLuc, mature PhoA was detected in the culture supernatant of the TS10 strain. Interestingly, not only the protein quantity but also the heterogeneity of the PhoA banding pattern increased significantly in the media of the genome-reduced strains. This was especially the case for PhoA produced by the IIG-Bs27-39 strain and further genome-reduced strains, where a prominent ladder-like pattern between ca. 50–100 kDa was observed, with two most prominent bands at 50 and 100 kDa (Figure 2B). Unlike the TrxA levels in the culture supernatants of strains producing GLuc, no significant changes in TrxA levels were detectable in the culture supernatants of strains with progressive genome reduction (Figure 2B), indicating comparable levels of cell lysis.

To gain insights into the activities of both expressed target proteins, GLuc and PhoA, we benchmarked each of them in a respective enzyme activity assay. Of note, in this case, the samples were not normalized for the OD_600_ to allow a comparison of the production of either target protein at the time of harvesting per volume of culture. The result of the luciferase assay for GLuc activity revealed that the amounts of active secreted protein increased steadily and significantly with each step in the genome reduction, leading to an increase of more than 3000-fold between *B. subtilis* TS10 and the IIG-Bs27-47-24 strain, and a 9-fold increase between the IIG-Bs27-31 and IIG-Bs27-47-24 strains (Figure 2C). The situation was different for the alkaline phosphatase activity in the culture supernatants, which was 1.5-fold increased between the TS10 and IIG-Bs27-39 strains. However, in contrast to GLuc, upon further genome reduction, a progressive decrease of PhoA activity with progressive genome reduction was observed (Figure 2D).

Interestingly, the OD_600_ of the investigated strains at the time of harvesting was variable, depending on the respective genomic reduction and the target protein that they produced. In the case of GLuc production, strain IIG-Bs27-39 reached the highest OD_600_, which was even higher than that of the reference strain TS10. Furthermore, the final OD_600_ of the two GLuc-producing strains with a larger genome reduction declined progressively. Yet, the IIG-Bs27-47-24 strain, which reached the lowest final OD_600_, produced the highest amount of active GLuc per volume of culture (Figure 2C). The situation was different for the strains expressing PhoA since, in this case, the IIG-Bs27-39 strain reached a much lower OD_600_ than the reference strain TS10 and the genome-reduced strains IIG-Bs27-31 and IIG-Bs27-47-14 (Figure 2D). On the other hand, the IIG-Bs27-39 strain produced the highest level of active PhoA. Thus, contrary to GLuc production, the further genome reduction in the IIG-Bs27-47-14 and IIG-Bs27-47-24 strains did not lead to increasing yields of active PhoA per volume of culture.

An important question was whether the GLuc produced by *B. subtilis* does indeed require disulfide bond formation for its enzymatic activity, as was previously shown for PhoA.^56^ Therefore, we incubated the cell and culture supernatant samples of GLuc-producing strain IIG-Bs27-39 with the reducing agent DTT to resolve any disulfide bonds in this protein. Upon DTT treatment, the GLuc activity in the culture supernatant samples vanished completely (Figure 2E). In contrast, the cellular samples showed no significant GLuc activity, neither before nor after incubation with DTT. Altogether, these observations demonstrate that the investigated genome-reduced *B. subtilis* strains have an increased capacity for GLuc secretion and that the activity of the secreted GLuc was strictly dependent on disulfide bond formation.

### Exoprotease Sensitivity of GLuc and PhoA

**Figure 3:**
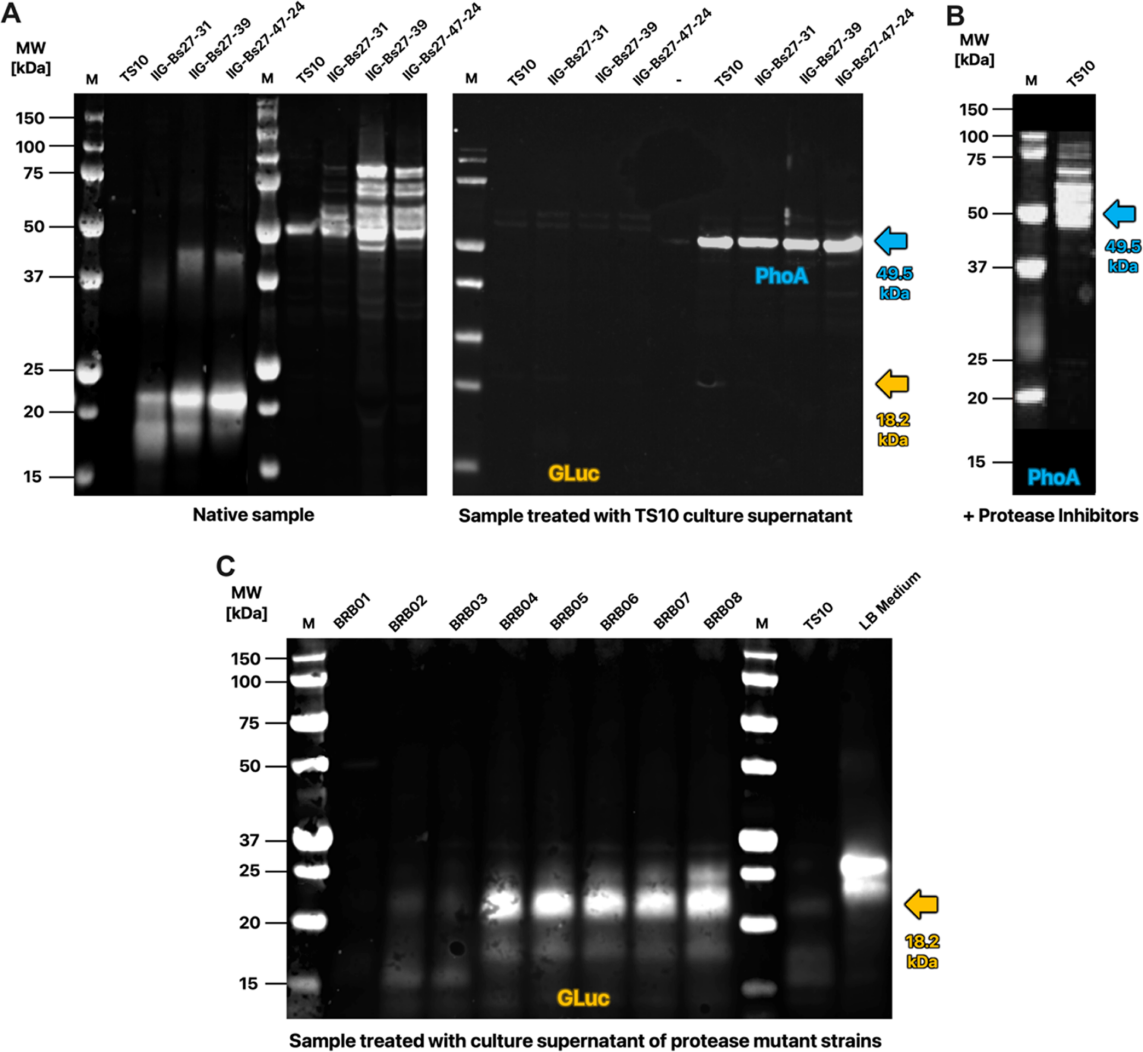
Protease sensitivity of GLuc and PhoA. (A) Culture supernatant samples from four different genome-reduced *B. subtilis* strains, producing either GLuc or PhoA, were incubated without addition (native samples) or with exoprotease-containing culture supernatant of the reference strain TS10. Subsequently, proteins in the samples were separated by LDS-PAGE and GLuc or PhoA were visualized by Western blotting using specific antibodies. The samples were normalized for the OD_600_ values of the respective strains. The respective arrows indicate the expected molecular size of mature GLuc and PhoA in kDa. (B) Western blot of PhoA secreted by the TS10 strain upon growth in a medium supplemented with protease inhibitors. (C) Western blot of culture supernatant samples of *B. subtilis* IIG-Bs27-39 expressing GLuc after incubation with culture supernatant samples of the protease-deleted *B. subtilis* 168 derivatives BRB01–BRB08, the TS10 reference strain (positive control), or fresh LB medium (negative control).

Previous studies have shown that the eight major exoproteases of *B. subtilis* can be highly detrimental to the production of heterologous proteins.^14^ Therefore, we decided to examine the impact of these exoproteases on the secretion of GLuc and *E. coli* PhoA by the genome-reduced *B. subtilis* strains IIG-Bs 27-31, IIG-Bs 27-39, and IIG-Bs 27-47-24. To this end, culture supernatant of the *B. subtilis* reference strain TS10 was incubated with equal volumes of spent growth media from the four genome-reduced strains producing either GLuc or PhoA. Western blotting analysis of GLuc showed that essentially all GLuc produced by the genome-reduced strains was degraded upon incubation with culture supernatant from the TS10 strain (Figure 3A). In contrast, Western blotting for PhoA secreted by the genome-reduced strains revealed that incubation with culture supernatant of the reference strain TS10 resulted in one single distinctive PhoA band with a size of 50 kDa, instead of the ladder-like pattern as observed before incubation (Figure 3A). For both GLuc and PhoA, the outcome of the incubation with culture supernatant of the TS10 strain was consistent with the results obtained for the production of either protein in the TS10 strain (Figure 2B). To verify that the disappearance of the ladder-like pattern observed for PhoA produced by the genome-reduced strains was due to protease activity in the medium of the TS10 strain, we assessed the secreted PhoA produced by the TS10 strain upon growth in the presence of protease inhibitors. As shown by Western blotting, this did indeed lead to the ladder-like banding pattern of PhoA (Figure 3B).

To determine which of the eight major *B. subtilis* exoproteases could be involved in the degradation of GLuc, we incubated the GLuc produced by the genome-reduced *B. subtilis* strain IIG-Bs27-39 with spent growth media from the *B. subtilis* strains BRB01–BRB08. The latter strains carry serial deletions of the eight major exoprotease-encoding genes.^14^ As shown in Figure 3C, complete degradation of GLuc occurred upon incubation with spent media of the BRB01 strain, which only lacks the *nprB* gene, as seen for TS10 with no protease gene deletion. Close to complete GLuc degradation was observed upon incubation with spent media of the BRB02 (lacking *nprB* and *aprE*) and BRB03 (lacking *nprB*, *aprE*, and *epr*) strains. Limited GLuc degradation was observed upon incubation with spent media of the BRB04, BRB05, BRB06, or BRB07 strains (all lacking the *bpr* gene), and GLuc degradation was further decreased upon incubation with spent medium of the BRB08 strain that lacks all major exoprotease genes. Nonetheless, compared to incubation with fresh LB medium, incubation of GLuc with spent media of the BRB04–08 strains still led to GLuc cleavage as evidenced by a GLuc band with higher mobility on LDS-PAGE (Figure 3C). It thus seems that several major exoproteases, especially Bpr and WprA, contribute to GLuc degradation but that the spent media of the genome-reduced strains still contain a proteolytic activity that is absent from the genome-reduced IIG-Bs27-39 strain. Altogether, it can be concluded that the absence of the genes for major exoproteases from the investigated genome-reduced *B. subtilis* strains contributes significantly to the yield of secreted GLuc by these strains, and to some extent the yield of secreted PhoA.

### TDOR Dependency of GLuc and PhoA Production in Genome-Reduced *B. subtilis*

**Figure 4:**
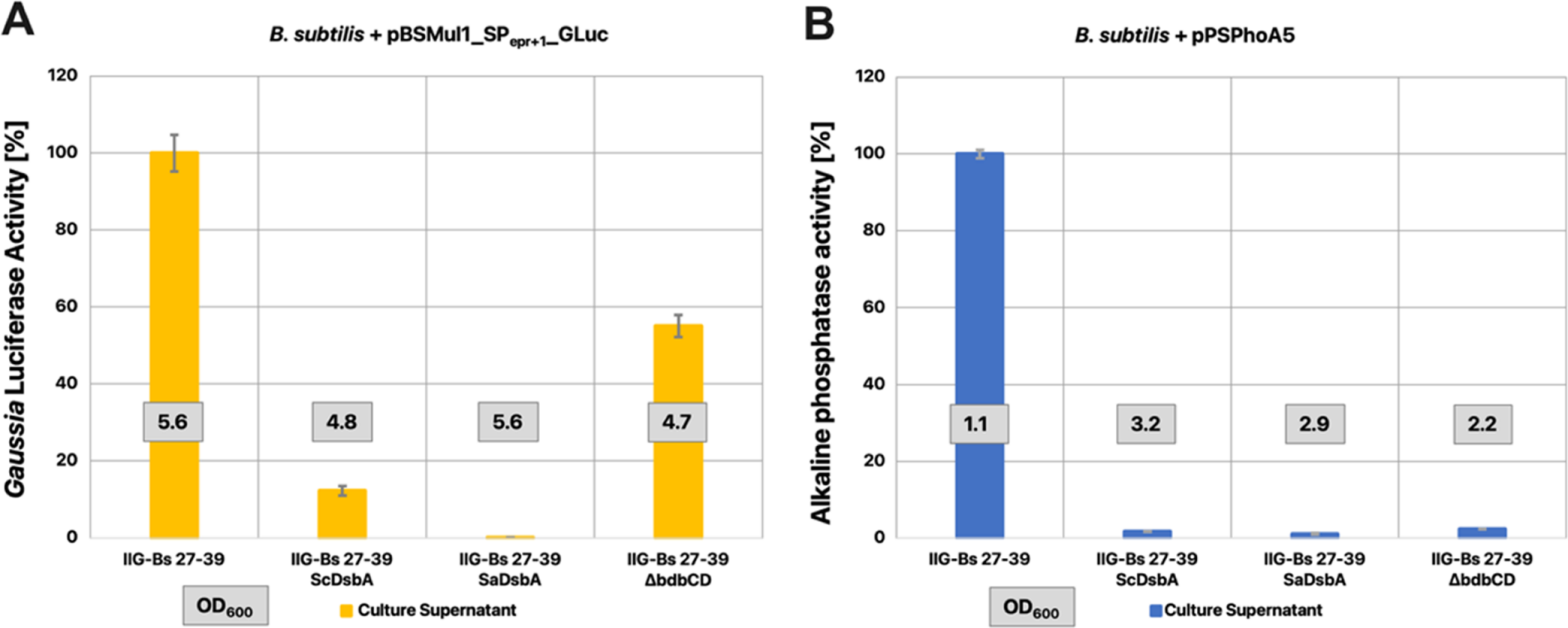
TDOR dependency of active GLuc or PhoA secretion in a genome-reduced *B. subtilis* background. Enzymatic activities of GLuc (A) or PhoA (B) in culture supernatant samples of the *B. subtilis* strain IIG-Bs27-39, and derivative strains either coexpressing SaDsbA or ScDsbA, or lacking the *bdbCD* genes. The GLuc and PhoA activities were measured as described in Figure 2. Boxed numbers indicate the respective OD_600_ of the expressing strain post fermentation. The measured data in relative light units (see Supporting Figure S2) was normalized, wherein the value measured for the reference strain IIG-Bs27-39 was set to 100%.

It was previously shown for *B. subtilis* 168 that the secretion of active *E. coli* PhoA is strongly dependent on the TDORs BdbC and BdbD, which are encoded by the *bdbDC* operon.^50^ We therefore investigated whether this was also the case for GLuc and PhoA in the genome-reduced background by deleting the *bdbC* and *bdbD* genes from strain IIG-Bs27-39. As shown in Figure 4, the secretion of active PhoA was negligible upon the deletion of the *bdbCD* genes from the IIG-Bs27-39 strain, which is fully consistent with the previously demonstrated BdbCD dependency of PhoA secretion. Interestingly, deletion of the *bdbCD* genes had a relatively moderate effect on the secretion of active GLuc, which was reduced by about 50% in the absence of BdbCD. This showed that in contrast to PhoA, the secretion of active GLuc is not strictly BdbCD-dependent.

In previous studies, it was also shown that the secretion of active *E. coli* PhoA could be improved by the coexpression of recombinant oxidative TDORs, namely, the DsbA proteins from either *Staphylococcus carnosus* (ScDsbA) or *Staphylococcus aureus* (SaDsbA).^25^ Notably, SaDsbA is one of the most potent thiol oxidases known.^57^ To ascertain whether this approach could also be beneficial for the secretion of active GLuc or PhoA in genome-reduced *B. subtilis* strains, we introduced the cassettes for ScDsbA or SaDsbA expression in the IIG-Bs27-39 strain. Unexpectedly, opposite to the positive effects previously observed in *B. subtilis* 168, introduction of either cassette completely abolished the secretion of active PhoA in the genome-reduced background (Figure 4B). Similarly, the introduction of the SaDsbA cassette completely abolished the secretion of active GLuc, while the introduction of the ScDsbA cassette strongly reduced active GLuc secretion (Figure 4A). Together, these observations show that GLuc and PhoA have different TDOR dependencies in a genome-reduced *B. subtilis* background.

## Discussion

Previous studies have shown that large-scale genome reduction confers novel traits to the *B. subtilis* cell factory that can be beneficial for recombinant protein production.^17,58,59^ In the present study, we wanted to explore whether this also applies to a protein with multiple disulfide bonds, since such proteins are notoriously difficult to produce in bacteria.^41^ The POI that we selected for this purpose was the luciferase of *Gaussia princeps* (GLuc), which has five disulfide bonds. Our results show that, unlike the reference strain TS10 with a full-size *B. subtilis* genome, genome-reduced *B. subtilis* strains are very well capable of secreting active GLuc.

To secrete active GLuc, we applied the SP of the exoprotease Epr of *B. subtilis* for the present studies. This turned out to be the most effective SP from a set of five tested SPs that were fused to GLuc immediately at the signal peptidase cleavage site or at the +1, + 2, or +3 residues of the respective mature proteins. Consistent with the notion that the secretion efficiency directed by a particular SP depends strongly on the target POI,^39^ we observed that only a few SP-GLuc fusions directed effective GLuc secretion. These were especially the SP_*wapA*+0_-, SP_*wapA*+1_-, and SP_*EPR*+1_-GLuc fusions. Previous investigations have shown that residues in the +1 to +3 region of the mature protein contribute to the protein secretion efficiency in *B. subtilis.*(39) However, since the SP_*wapA*+0_-GLuc fusion performed better than the SP_*wapA*+1_-GLuc fusion, inclusion of the +1 residue in the SP-GLuc fusion does not necessarily lead to the highest secretion efficiency. Interestingly, in contrast to the levels of secreted GLuc, which varied substantially, we observed that the GLuc-secreting bacteria contained fairly similar cellular levels of active GLuc. Since the bacterial cytoplasm is a reducing environment and GLuc requires disulfide bonding for its activity, we assume that the cell-associated active GLuc molecules have been translocated across the membrane and reside at the membrane–cell wall interface or in the cell wall, which are oxidizing environments. If this is the case, the fact that the levels of cell-associated GLuc are very similar would suggest that particular sites in the bacterial cell envelope need to be saturated with GLuc before secretion occurs. However, this needs to be further investigated.

All genome-reduced *B. subtilis* strains that we tested in our present study showed GLuc secretion directed by SP_*EPR*+1_, in contrast to the reference strain TS10. Interestingly, GLuc secretion improved progressively with increasing genome reduction. Thereby, no increase but rather a slight decrease in cell lysis was observed. This implies that the increased GLuc concentration in the culture supernatant is, in fact, caused by active secretion and not by cell lysis. Concomitantly with increasing genome reduction, we observed fewer GLuc degradation products and a major shift to the secretion of the mature-sized GLuc protein. This implies that the successive steps in genome reduction led to reduced proteolysis of secreted GLuc. Indeed, exposure of the mature secreted GLuc to exoproteases secreted by reference strain TS10 resulted in rapid and complete GLuc degradation, showing that this protein is intrinsically protease-sensitive. Furthermore, incubation of the secreted GLuc with spent growth media of strains with serially deleted exoproteases showed that especially the exoprotease Bpr, and to a lesser extent also WprA, contributes to GLuc degradation. Still, even upon deletion of all eight major exoproteases, the culture supernatant of the BRB08 strain contained as yet unidentified proteolytic activity that led to GLuc cleavage.

While it is known that deletion of *Bacillus* exoproteases can increase the yields of secretory proteins many-fold, the massive performance boost in the secretion of active GLuc among the increasingly genome-reduced strains is remarkable. After the already steep 336-fold activity increase between the TS10 and IIG-Bs27-31 strains, the additional 9.4-fold increase between the IIG-Bs27-31 and IIG-Bs27-47-24 strains was particularly surprising. Judged by the results from our previous study on the secretion of a difficult-to-produce staphylococcal protein by the IIG-Bs27-47-24 strain,^59^ the massive increase of more than 3000-fold overall in secretory GLuc production may also originate from other beneficial traits of this strain. These include an improved capacity for translation, increased levels of Sec secretion machinery components and chaperones, increased levels of the quality control proteases HtrA and HtrB that degrade misfolded proteins, and decreased competition for the Sec pathway by other Sec-dependently secreted proteins of which the genes had been removed.

At present, we do not know exactly how the enhanced translational efficiency is brought about in genome-reduced *B. subtilis* strains. One possibility is that this relates to an upregulation of ribosomal proteins,^59^ but it could also be a consequence of the reduced number of translatable mRNAs.^17^ However, it should be noted that genetic regulation is complex and takes place at multiple levels. Therefore, the size of a genome or the number of encoded genes does not directly determine the number of translatable mRNAs. In terms of genome reduction starting from *B. subtilis* 168, the genome of the IIG-Bs14 strain (not tested in this study) was reduced by ca. 11%. However, the genes deleted from this strain all belong to prophage regions and/or code for *Bacillus* toxins that are not translated under normal laboratory conditions^47^ either due to lack of transcription, small RNA interference, or fast selective mRNA degradation.^60^ Compared to the 168 strain, the genome-reduced strains IIG-Bs27-31, IIG-Bs27-39, IIG-Bs27-47-24, and the mini*Bacillus* strain PG10 lack 19, 28, 31, and 39% of the genome, respectively. Interestingly, the presently used strain IIG-Bs27-31, and all genome-reduced strains further down in the phylogeny, lack the genes for the sigma factors SigE, SigF, and SigG, as well as multiple of their regulators. This should lead to a further decrease in the number of translatable mRNAs. In consequence, this could increasingly limit the levels of translational competition in the IIG-Bs27-31, IIG-Bs27-39, and IIG-Bs27-47-24 strains than one may solely anticipate based on the level of genome reduction. On the other hand, starting from the IIG-Bs27-31 strain, the activity of GLuc was increased by 2.4-fold in the IIG-Bs27-39 strain, 4.5-fold in the IIG-Bs27-47-14 strain, and 9.4-fold in the IIG-Bs27-47-24 strain. These effects are unproportionally higher than what we would have expected solely based on decreased levels of mRNAs competing for translation. Hence, there are most likely more factors involved in the observed yield increase of active GLuc in our study, which could be based on an enhanced capacity for Sec-dependent translocation and increased post-translocation protein folding and quality control.

For PhoA of *E. coli*, a less drastic increase of secreted active protein was observed when expressed in the genome-reduced strains. Constant levels of observed cell lysis confirmed that the increased levels of PhoA detected in the culture supernatant were not caused by cell lysis but by active PhoA secretion. In particular, the activity of secreted PhoA was 1.5-fold increased in the IIG-Bs27-39 strain compared to that in the reference strain TS10. Interestingly, the PhoA secreted by the genome-reduced strains did not migrate on LDS-PAGE as a single band, but rather it showed a ladder-like banding pattern with prominent bands of ∼50 and ∼100 kDa. The expected size of *E. coli* PhoA is ∼50 kDa, which conforms to the PhoA band detectable in the culture supernatant of the TS10 strain. However, to enhance PhoA secretion, this protein was fused to the SP and pro-peptide from a *Staphylococcus hyicus* lipase, where the pro-peptide also has a predicted molecular weight of ∼50 kDa.^61^ Hence, the resulting pro-PhoA has a molecular weight of ∼100 kDa, which implies that the intermediate PhoA-specific protein bands relate to proteolytic cleavage events within the pro-peptide. In *S. hyicus*, the lipase pro-peptide is processed by the site-specific cell wall-associated metalloprotease ShpII.^62^ In *B. subtilis*, this function is apparently taken over by one or more exoproteases, leading to complete removal of the pro-peptide in the TS10 strain. Upon consecutive reduction of extracellular proteolysis in the genome-reduced strains, partial pro-peptide proteolysis occurs, leading to the ladder-like banding pattern of secreted pro-PhoA species. Such a banding pattern was previously also described for the same pro-PhoA product upon downregulated expression of *trxA* and/or coexpression of a staphylococcal DsbA.^25^ Concomitantly, the extracellular PhoA activity increased around 3-fold (activity normalized to OD_600_), which is around the same increase as presently observed in the IIG-Bs27-39 strain (around 3-fold if normalized to OD_600_). Of note, while the IIG-Bs27-39 strain generally reached the highest OD_600_ of all strains tested in our study, the final OD_600_ of the IIG-Bs27-39 secreting active *E. coli* PhoA was about 2-fold lower than that of the TS10 strain. This indicates that the secretion of functional PhoA was somewhat detrimental to the growth of *B. subtilis*.

The intricate interdependencies of extracytoplasmic thiol oxidation and cross-membrane electron flow in *B. subtilis*, including all of the factors involved, are not fully understood so far. However, the main TDORs of *B. subtilis* have been identified. Previous studies have shown that thiol oxidation in exported proteins requires primarily the membrane-bound thiol oxidase BdbD, which forms a functional pair with the quinone oxidoreductase BdbC.^63^ BdbC is believed to reoxidize BdbD and donate electrons to membrane-embedded quinones for further transfer to oxygen. The SPβ prophage-encoded quinone oxidoreductase BdbB, a paralogue of BdbC, is specifically required for extracytoplasmic disulfide bond formation in the SPβ prophage-encoded bacteriocin sublancin 168.^64^ Accordingly, the dispensable *bdbB* gene was lost already in the early stages of genome reduction of the presently investigated strains.^16^ On the other hand, *B. subtilis* has a pathway for extracytoplasmic thiol reduction, which is based on the membrane-embedded CcdA protein and the membrane-bound extracytoplasmic thiol-reductases ResA and StoA. The latter two reductases are, respectively, required for cytochrome c biogenesis and sporulation.^65,66^ Since the *bdbCD*, *ccdA*, *resA*, and *stoA* genes are still present in the here investigated genome-reduced strains, these strains seem to avail of the “hardware” required for thiol oxidation, disulfide bond reduction, and potentially disulfide bond isomerization. Our present results show that in the genome-minimized strain IIG-Bs27-39, the secretion of active PhoA is BdbCD-dependent, which is consistent with previous observations in the *B. subtilis*-type strain 168.^24^ At present, it is, however, unclear why GLuc is only partially BdbCD-dependent. Conceivably, disulfide bond formation in this protein could also be catalyzed by other, yet unidentified, extracytoplasmic TDORs or by free low-molecular-weight thiols. Here it is noteworthy that previous studies showed that reduced GLuc can recover its activity by chemical reoxidation in a glutathione redox buffer, even in the absence of isomerase activity.^67^ This could be explained by the specific two-domain structure of GLuc, which thermodynamically favors intradomain folding prior to interdomain folding and thiol oxidation. Such a folding mechanism would determine the sequential order of dithiol oxidation and minimize possible mismatches in disulfide bonding. Clearly, this would be beneficial for GLuc folding as there are theoretically 975 different ways to arrange this protein’s 10 cysteines into 5 disulfide bonds.^45^ On the contrary, *E. coli* PhoA has four cysteines that can be arranged in three different ways into two disulfide bonds, a process that is strongly dependent on the activity of thiol oxidases both in *B. subtilis* and *E. coli.*(63)

Lastly, in view of the many cysteine residues in GLuc, it is conceivable that overexpression of very strong oxidases, like the staphylococcal DsbA proteins, is counterproductive for the secretion of GLuc in *B. subtilis*, as was observed in the present study. In such a situation, there would be a high(er) requirement for isomerase activity to reshuffle incorrectly formed disulfide bonds. Such isomerase activity can be provided by an interplay of disulfide-reducing and thiol-oxidizing TDORs.^24^ This draws attention to the disulfide reductases ResA and StoA, which could contribute to disulfide bond isomerization but may not be sufficiently active under the tested conditions to reshuffle wrongly formed disulfide bonds in GLuc. A similar explanation could be entertained to explain the presently observed negative effect of DsbA coexpression on the secretion of active PhoA. Possibly, the delicate balance between oxidizing and reducing TDORs has shifted toward oxidizing TDORs, like BdbCD, due to the genome reduction, which would make coexpression of DsbA counterproductive. In addition, it is conceivable that the expression of TDOR-encoding genes is altered in the presently investigated genome-reduced strains. For instance, these strains lack the genes for the early and late sporulation-specific sigma factors SigE and SigG, which have been implicated in the expression of *bdbDC* and *stoA*.^68^ Moreover, a possible limitation in the activity of disulfide reductases like ResA or StoA in genome-reduced strains could potentially be caused by reduced expression of the respective genes or a lowered availability of reducing equivalents due to metabolic rearrangements. Indeed, there is evidence for both options as a previous proteomics analysis revealed changes in amino acid and nitrogen metabolism in the IIG-Bs27-47-24 strain, as well as an altered oxidative stress response.^59^

## Conclusions

Our present study shows that genome-reduced “midi*Bacillus*” strains offer excellent opportunities for the expression of difficult-to-produce POIs, even those with multiple disulfide bonds. This is underscored by the more than 3000-fold increase that we observed for the production of active GLuc in the IIG-Bs27-47-24 strain. Our results show that this effective secretion of GLuc relates strongly to the protease deficiency of the genome-minimized strains but not exclusively, as we have previously shown that such strains have an enhanced capacity for translation and protein secretion via the Sec pathway. Our present study also identifies some possible shortcomings of the genome-reduced strains. For instance, they were not sufficiently able to proteolytically remove the pro-peptide from the secreted PhoA protein, and in most cases, they grew to lower cell densities. The latter may actually also be an advantage for biotechnological applications because the objective here is not to produce biomass but rather the POI of interest. Lastly, our study suggests that the balance between thiol-oxidizing and -reducing activities in genome-reduced strains could be altered. This certainly leaves room for future investigations in order to further enhance the capacity for the production of POIs with multiple disulfide bonds. Nonetheless, despite some potential current shortcomings, we do advocate the use of genome-reduced *B. subtilis* strains as chassis for the production of difficult-to-produce target proteins.

## Data Availability

All data generated or analyzed during this study are included in this published article and its Supporting Information files. Plasmid maps and sequences have been deposited on Zenodo with the DOI 10.5281/zenodo.8125543 and are available at 10.5281/zenodo.8125543.

## Supporting Information Available

Signal peptide screening for GLuc secretion by *B. subtilis* (Figure S1); benchmark of different genome-minimized strains producing GLuc or PhoA (Figure S2); oligonucleotides used in this study (Table S1); and the nonprocessed read-out data of the GLuc and PhoA activity assays in Figure 1, 2, and 4 (PDF)
