# Supplementary material for: Let There Be Light: Genome Reduction Enables Bacillus subtilis to Produce Disulfide-Bonded Gaussia Luciferase
Source: ACS Synth Biol. 2023 Nov 27;12(12):3656–68. doi: 10.1021/acssynbio.3c00444 (PMC10729301; doi:10.1021/acssynbio.3c00444)
Supplement: Supplementary file 1 — sb3c00444_si_001.pdf [file sb3c00444_si_001.pdf]

Supplementary Materials

Let There Be Light: Genome-reduction Enables *Bacillus subtilis* to Produce Disulfide-bonded *Gaussia* Luciferase

Tobias Schilling[1], Borja Ferrero-Bordera [2], Jolanda Neef[1], Sandra Maaβ[2], Dörte Becher[2], and Jan Maarten van Dijl[1]*

[1] University of Groningen, University Medical Center Groningen, Department of Medical Microbiology, Hanzeplein 1, P.O. Box 30001, 9700RB Groningen, The Netherlands

[2] University of Greifswald, Institute of Microbiology Department of Microbial Proteomics, D-17489 Greifswald, Germany

* To whom correspondence should be addressed: Tel. +31503615187; Email: j.m.van.dijl01@umcg.nl

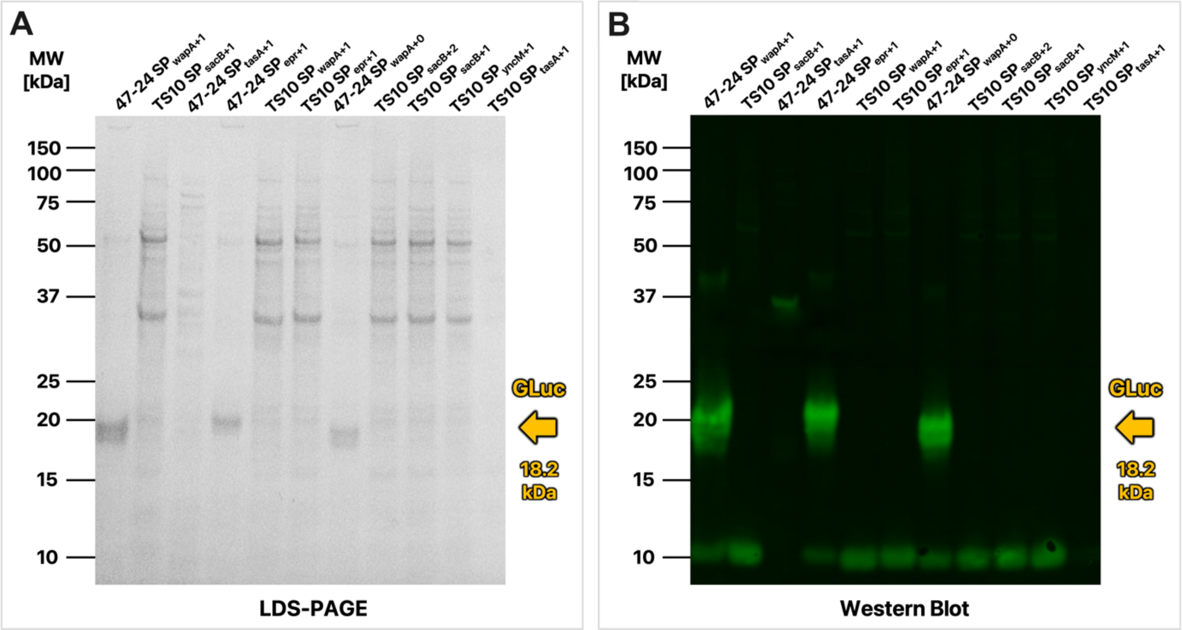

Supplementary Figure S1: Signal peptide screening for GLuc secretion by *B. subtilis*. Cells of *B. subtilis* TS10 or IIG-Bs27-47-24 producing GLuc with N-terminal signal peptide fusions as indicated were separated from the growth medium by centrifugation after 16 h of growth in LB medium at 37°C. Subsequently, proteins in the supernatant fractions were separated by LDS-PAGE, and visualized by staining with Coomassie (**A**) or by Western blotting using specific antibodies against GLuc (**B**). The expected position of mature GLuc in the LDS-PAGE and Western blot is indicated with an arrow.

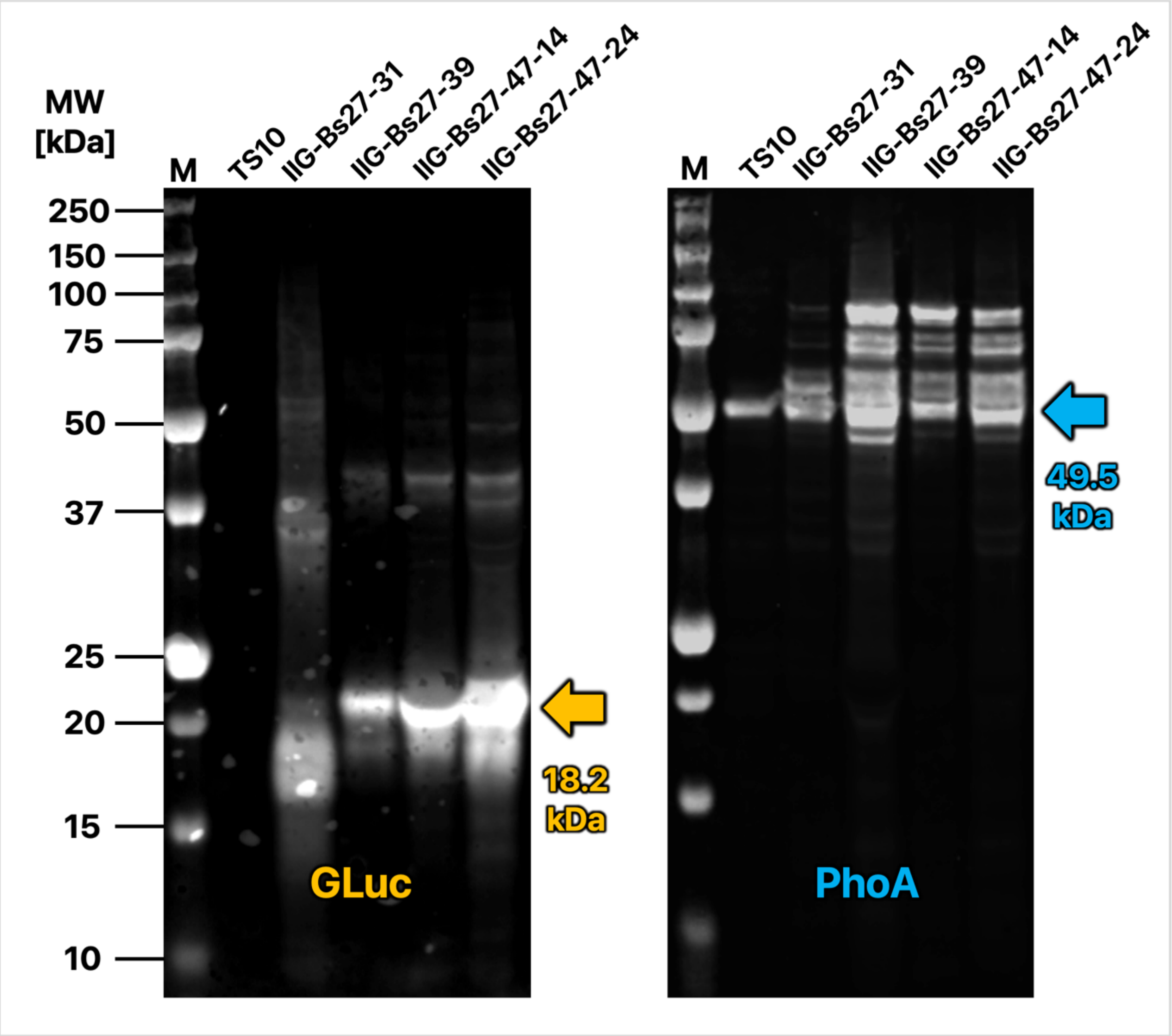

Supplementary Figure S2: Benchmark of different genome-minimized strains producing GLuc or PhoA. Culture supernatant samples from four different genome-minimized *B. subtilis* strains and the reference strain TS10, producing either GLuc or PhoA, were separated by LDS-PAGE and analyzed Western blotting with specific antibodies against GLuc or PhoA, respectively. As opposed to the blots shown in Fig. 2B in the main manuscript, these blots were not incubated with an antibody against TrxA to visualize possible cell lysis.

Supplementary Table S1: Oligonucleotides used in this study.

Oligonucleotide Name	Sequence
TS101	tacgGATAAATAGGAAATGAGACT
TS102	AAACAGTCTCATTTCCTATTTATC
TS103	CGACGGCCAACGAGGtaatatgttacatggccatcaaacat
TS104	gctagcggcaaattccgccatgatgt
TS105	ggaatttgccgctagctttttatttttaaaaaattg
TS106	atattgggtgggaggatttcgtgccg
RH001	AGCTTAGGCCCAGTCGAAAG
RH002	CAGCTAGGAGGTGACTGAAG
RH003	ACCGAGCGTTCTGAACAAATCC
pBSMul1.fw	GATATCTCCTCCTTATTATGTAAATCGCTCC
pBSMul1.rev	TCTAGAGGTCGAAATTCACCTCGAAAGC
GLuc JN.fw	TAAGGAGGAGATATCAAACCAACTGAAAAC
GLuc JN.rv	ATTTCGACCTCTAGATTAATCACCACCGGCACCCTTTATTTTGTCCACTTG
eprJN.fw	GTTTTCAGTTGGTTTGCTGTTTTGCGCATGAGCGAGAGGgcctatgg
eprJN.rv	CCTCTCGCTCATGCGAAACCAACTGAAAAC
epr+1AAJN.rv	GTTTTCAGTTGGTTTCGCATGAGCGAGAGGgcctatgg
epr+2AAJN.rv	GTTTTCAGTTGGTTTGTTTTGCGCATGAGCGAGAGGgcctatgg
epr+3AAJN.rv	GTTTTCAGTTGGTTTGCTGTTTTGCGCATGAGCGAGAGGgcctatgg
epr-GLucJN+0AA.fw	CCTCTCGCTCATGCGAAACCAACTGAAAAC
epr-GLucJN+1AA.fw	CCTCTCGCTCATGCGCAAAAACCAACTGAAAACAATGAAGATTTCAACATTGTAGCTG TAGC
epr-GLucJN+2AA.fw	CCTCaTCGCTCATGCGCAAAACAAACCAACTGAAAACAATGAAGATTTCAACATTGTA GCTGTAGC
epr-GLucJN+3AA.fw	CCTCTCGCTCATGCGCAAAACAGCAAACCAACTGAAAACAATGAAGATTTCAACATT GTAGCTGTAGC
WapAJN.fw	TAAGGAGGAGATATCATGAAAAAAAGAAAGAGGCGAAACTTTAAAAGGTTCATTGC AGC
wapA+0AAJN.rv	CATTGTTTTCAGTTGGTTTTGCTAGTACATCGGCTGGCACTAATGAAATCATTAAAGC C
wapA+1AAJN.rv	CATTGTTTTCAGTTGGTTTTTTTGCTAGTACATCGGCTGGCACTAATGAAATCATTAAA GCC
wapA+2AAJN.rv	CATTGTTTTCAGTTGGTTTAGATTTTGCTAGTACATCGGCTGGCACTAATGAAATCATT AAAGCC

wapA+3AAJN.rv	CATTGTTTTCAGTTGGTTTTGTAGATTTTGCTAGTACATCGGCTGGCACTAATGAAATCATTAAAGCC
wapA-GLuc+0JN.fw	GCCAGCCGATGTACTAGCAAAACCAACTGAAAACAATGAAGATTTCAACATTGTAGCTGTAGCTAGC
wapA-GLuc+1JN.fw	GCCAGCCGATGTACTAGCAAAAAAACCAACTGAAAACAATGAAGATTTCAACATTGTAGCTGTAGCTAGC
wapA-GLuc+2JN.fw	GCCAGCCGATGTACTAGCAAAATCTAAACCAACTGAAAACAATGAAGATTTCAACATTGTAGCTGTAGCTAGC
wapA-GLuc+3JN.fw	GCCAGCCGATGTACTAGCAAAATCTacaAAACCAACTGAAAACAATGAAGATTTCAACATTGTAGCTGTAGCTAGC
sacBJN.fw	TAAGGAGGAGATATCATGAACATCAAAAAGTTTGCAAAACAAGCAACAGTATTAACCTTTACTaccgc
sacBJN.rv	TCATTGTTTTCAGTTGGTTTCGCAAACGCTTGAGTTGCGCCTCC
sacB+1AAJN.rv	TCATTGTTTTCAGTTGGTTTTTTCGCAAACGCTTGAGTTGCGCCTCC
sacB+2AAJN.rv	TCATTGTTTTCAGTTGGTTTTTCTTTCGCAAACGCTTGAGTTGCGCCTCC
sacB+3AAJN.rv	TCATTGTTTTCAGTTGGTTTCGTTTCTTTCGCAAACGCTTGAGTTGCGCCTCC
sacB_GLucJN.fw	GCAACTCAAGCGTTTGCGAAACCAACTGAAAACAATGAAGATTTCAACATTGTAGCTGTAGCTAGC
sacB_GLuc+1JN.fw	GCAACTCAAGCGTTTGCGaaaAAACCAACTGAAAACAATGAAGATTTCAACATTGTAGCTGTAGCTAGC
sacB_GLuc+2JN.fw	GCAACTCAAGCGTTTGCGAAAGAAAAACCAACTGAAAACAATGAAGATTTCAACATTGTAGCTGTAGCTAGC
sacB_GLuc+3.fw	GCAACTCAAGCGTTTGCGAAAGAAACGAAACCAACTGAAAACAATGAAGATTTCAACATTGTAGCTGTAGCTAGC
yncMJN.fw	CATAATAAGGAGGAGATATCATGGCGAAACCACTATCAAAAGGGGGAATTTTGG
yncM.rv	CTTCATTGTTTTCAGTTGGTTTTGCTTTTGCGACTTGAGCGTCTGCCG
yncM+1JN.rv	CTTCATTGTTTTCAGTTGGTTTTGCTGCTTTTGCGACTTGAGCGTCTGCCG
yncM+2JN.rv	CTTCATTGTTTTCAGTTGGTTTGGATGCTGCTTTTGCGACTTGAGCGTCTGCCG
yncM+3JN.rv	CTTCATTGTTTTCAGTTGGTTTCTCGGATGCTGCTTTTGCGACTTGAGCGTCTGCCG
yncM_GLucJN.fw	CAAGTCGCAAAAGCAAAACCAACTGAAAACAATGAAGATTTCAACATTGTAGCTGTAGC
yncM_GLuc+1AAJN.fw	CAAGTCGCAAAAGCAGCAAAACCAACTGAAAACAATGAAGATTTCAACATTGTAGCTGTAGC
yncM_GLuc+2AAJN.fw	CAAGTCGCAAAAGCAGCATCCAAACCAACTGAAAACAATGAAGATTTCAACATTGTAGCTGTAGC
yncM_GLuc+3AAJN.fw	CAAGTCGCAAAAGCAGCATCCGAGAAACCAACTGAAAACAATGAAGATTTCAACATTGTAGCTGTAGC
tasAJN.fw	TAAGGAGGAGATATCATGGGTATGAAAAAGAAATTGAGTTTAGGAGTTGCTTCTGCA

	GC
tasAJN.rv	TGTTTTCAGTTGGTTTTGCCCATGTTCCTCCTCCAACTAAAGCTAATCCTAG
tasA+1AAJN.rv	TGTTTTCAGTTGGTTTTGCTGCCCATGTTCCTCCTCCAACTAAAGCTAATCCTAG
tasA+2AAJN.rv	TGTTTTCAGTTGGTTTAAATGCTGCCCATGTTCCTCCTCCAACTAAAGCTAATCCTAG
tasA+3AAJN.rv	TGTTTTCAGTTGGTTTGTTAAATGCTGCCCATGTTCCTCCTCCAACTAAAGCTAATCCTAG
tasA_GLucJN.fw	GGAGGAACATGGGCAAAACCAACTGAAAACAATGAAGATTTCAACATTGTAGCTGTAGCTAGC
tasA_GLuc+1AAJN.fw	GGAGGAACATGGGCAGCAAAACCAACTGAAAACAATGAAGATTTCAACATTGTAGCTGTAGCTAGC
tasA_GLuc+2AAJN.fw	GGAGGAACATGGGCAGCATTTAAACCAACTGAAAACAATGAAGATTTCAACATTGTAGCTGTAGCTAGC
tasA_GLuc+3AAJN.fw	GGAGGAACATGGGCAGCATTTAACAAACCAACTGAAAACAATGAAGATTTCAACATTGTAGCTGTAGCTAGC
pBSMullcol.fw	GTGGTTGCCGGAAAGAGCGAAAATGCCTC
pBSMullcol.rev	GGAGCCTTTAATTGTATCGGTTTATCAGCTTGC

Supplementary Table S2: Non-processed read-out data of the GLuc and PhoA activity assays in Figures 1, 2 and 4.

Supplementary Data for Fig. 1: GLuc Activity Assay (Light Emission at 485 nm)

Signal peptide	Cell Pellet Read 1 [RLU]	Cell Pellet Read 2 [RLU]	Cell Pellet Read 3 [RLU]	Supernatant Read 1 [RLU]	Supernatant Read 2 [RLU]	Supernatant Read 3 [RLU]
[Blank]	5	8	13	5	8	13
SP_{WapA+0}	3547	4174	3920	47106	53303	47537
SP_{WapA+2}	5129	4910	5029	16016	16783	16708
SP_{TasA+1}	3008	2990	3399	287	297	374
SP_{Epr+1}	2807	2989	3354	57346	55438	60947

Supplementary Data for Fig. 2C: GLuc Activity Assay (Light Emission at 485 nm)

B. subtilis Strain	Cells Read 1 [RLU]	Cells Read 2 [RLU]	Cells Read 3 [RLU]	Supernatant Read 1 [RLU]	Supernatant Read 2 [RLU]	Supernatant Read 3 [RLU]	OD_{600}
[Blank]	4	11	9	26	25	63	-
TS10	48	52	53	9566	10717	10351	3.75
IIG-Bs 27-31	341	332	316	25561	24509	24232	3.42
IIG-Bs 27-39	622	583	689	44854	46494	46609	4.10
IIG-Bs 27-47-14	874	793	832	82518	97991	107080	1.97
IIG-Bs 27-47-24	1439	1396	1554	26	25	63	1.54

Supplementary Data for Fig. 2D: PhoA Activity Assay (Optical Density at 405 nm)

B. subtilis Strain	Cell Pellet Read 1	Cell Pellet Read 2	Cell Pellet Read 3	Supernatant Read 1	Supernatant Read 2	Supernatant Read 3	OD_{600}
[Blank]	0	0	0	0	0	0	-
TS10	1.0522	0.9207	1.0928	1.2783	1.207	1.2748	3.825
IIG-Bs 27-31	0.9749	1.1705	1.0493	1.3828	1.4115	1.4034	3.0965
IIG-Bs 27-39	0.8755	0.6474	0.8746	1.8981	1.9098	1.9239	1.8915
IIG-Bs 27-47-14	1.0272	1.1473	0.9891	1.1623	1.1616	1.1758	3.034
IIG-Bs 27-47-24	0.4032	0.4138	0.3974	0.8829	0.8826	0.8841	1.313

Supplementary Data for Fig. 2E: GLuc Activity Assay (Light Emission at 485 nm)

Sample Type	Cells Read 1 [RLU]	Cells Read 2 [RLU]	Cells Read 3 [RLU]	Supernatant Read 1 [RLU]	Supernatant Read 2 [RLU]	Supernatant Read 3 [RLU]
[Blank]	10	5	8	20	22	14
Native	245	3412	2844	173198	187108	196076
DTT treated	224	298	250	20	22	14

Supplementary Data for Fig. 4A: GLuc Activity Assay (Light Emission at 485 nm)

B. subtilis Strain IIG-Bs27-39	Cells Read 1 [RLU]	Cells Read 2 [RLU]	Cells Read 3 [RLU]	Supernatant Read 1 [RLU]	Supernatant Read 2 [RLU]	Supernatant Read 3 [RLU]	OD_{600}
[Blank]	4	5	11	7	6	6	-
WT	1495	1606	1703	65781	60791	66323	5.56
*Sc*DsbA	302	327	342	7340	7550	8802	4.77
*Sc*DsbA	172	199	190	65	63	45	5.56

ΔBdbCD	939	956	1026	37435	33937	34845	4.70
**Supplementary Data for Fig. 4B: PhoA Activity Assay (Optical Density at 405 nm)**							
***B. subtilis* Strain IIG-Bs27-39**	**Cells Read 1**	**Cells Read 2**	**Cells Read 3**	**Supernatant Read 1**	**Supernatant Read 2**	**Supernatant Read 3**	**OD_{600}**
[Blank]	-	-	-	0.082	0.081	0.081	-
WT	-	-	-	0.999	1.019	0.999	1.11
*Sc*DsbA	-	-	-	0.097	0.097	0.098	3.16
*Sc*DsbA	-	-	-	0.09	0.09	0.094	2.93
ΔBdbCD	-	-	-	0.102	0.103	0.103	2.16

RLU, relative light units
